# Daily intake of 30 mg duloxetine is effective in decreasing premature ejaculation severity: a prospective randomized placebo-controlled cross over clinical trial

**DOI:** 10.1186/s12610-023-00210-1

**Published:** 2023-12-05

**Authors:** Adham Zaazaa, Mohamed Nasr Eldin, Sameh Fayek GamalEl Din, Ashraf Zeidan, Mohamed Yassin Mohamed Saleh, Ahmed Adel, Mohamed Shokr

**Affiliations:** 1 Department of Andrology, Sexology & STDs – Kasr AlAiny Faculty of Medicine, Cairo University, Al-Saray Street, El Manial, Cairo, 11956 Egypt; 2 Department of Psychiatry Medicine-Kasr AlAiny Faculty of Medicine, Cairo University, Cairo, Egypt; 3 Faculty of Medicine, 6th October University, Giza, Egypt

**Keywords:** Lifelong premature ejaculation, Acquired premature ejaculation, Duloxetine, Sexual quality of life-men version

## Abstract

**Background:**

Premature ejaculation (PE) is considered to be the most common male sexual disorder affecting 20% to 66% of sexually active men. Most of the patients had already tried on demand dapoxitine with no improvement. We aimed in the current study to assert the efficacy and safety profile of daily intake of 30 mg duloxetine in treating patients with lifelong premature ejaculation (LPE) as well as patients with acquired premature ejaculation (APE).

**Results:**

The current study showed significant improvement in intravaginal ejaculatory latency time (IELT) after intake of duloxetine. All participants had a median Arabic index of premature ejaculation (AIPE) of 26, median IELT of 180 s, median male sexual quality of life (SQOL) of 43 after being treated with duloxetine (*p* value < 0.001 for all). While median AIPE after placebo was 19, median IELT after placebo was 60 s and median male SQOL after placebo was 21. Paired comparison of AIPE, IELT (Secs), inter quartile range (IQR) and male SQOL in group (A) patients at baseline and after duloxetine intake showed statistically significant improvement among treated patients (*p* values < 0.001 for all). Paired comparison of AIPE, IELT (Secs), IQR and male SQOL in group (A) patients at baseline and after placebo treatment showed no significant improvement of male SQOL. Furthermore, AIPE and IELT returned to baseline scores after discontinuation of duloxetine (*p* values 0.729; 0.892, respectively). Paired comparison of AIPE, IELT (Secs), IQR and male SQOL in group (B) patients at baseline and after placebo treatment showed almost same scores of patients in group (A) who received placebo for 2 months after a 2 month washout period (*p* values 1.000 for all).

Paired comparison of AIPE, IELT (Secs), IQR and male SQOL in group (B) patients at baseline and after duloxetine treatment showed statistically significant improvement among all treated patients (*p* values < 0.001 for all).

**Conclusion:**

Duloxetine is an effective drug for treatment of LPE and APE patients. Further, larger studies are needed to compare duloxetine to different known therapeutic modalities for PE to assert it’s efficacy and superiority.

## Résumé

**Contexte:**

L’éjaculation précoce (EP) est considérée comme le trouble sexuel masculin le plus courant, touchant 20% à 66% des hommes sexuellement actifs. La plupart des patients avaient déjà essayé la dapoxitine à la demande sans amélioration. Dans la présente étude, nous avons cherché à déterminer l’efficacité et l’innocuité de l’apport quotidien de 30 mg de duloxétine comme traitement de patients atteints d’éjaculation précoce de longue durée et de patients atteints d’éjaculation précoce acquise.

**Résultats:**

La présente étude a montré une amélioration significative du temps de latence éjaculatoire intravaginal (IELT) après la prise de duloxétine. Tous les participants avaient un indice médian de l’éjaculation précoce (AIPE) de 26, un IELT médian de 180 secondes, et une médiane de la qualité de vie sexuelle masculine (SQOL) de 43 après avoir été traités par la duloxétine (*p* < 0,001 pour tous). Après placebo, l’AIPE médian était de 19, l’IELT médian de 60 secondes et la médiane de la SQOL masculine de 21. La comparaison appariée de l’AIPE, de l’IELT, de l’intervalle interquartile (IQR) et de la SQOL masculine chez les patients du groupe (A) au départ et après la prise de duloxétine a montré une amélioration statistiquement significative chez les patients traités (*p* < 0,001 pour tous). La comparaison appariée de l’AIPE, de l’IELT, de l’IQR et de la SQOL masculine chez les patients du groupe (A) au départ et après traitement par placebo n’a montré aucune amélioration significative de la SQOL masculine. De plus, l’AIPE et l’IELT sont revenus aux scores de base après l’arrêt de la duloxétine (respectivement p 0,729; 0,892).

La comparaison appariée de l’AIPE, de l’IELT, de l’IQR et de la SQOL masculine chez les patients du groupe (B) au départ et après traitement par placebo a montré presque les mêmes scores que les patients du groupe (A) qui ont reçu un placebo pendant 2 mois après une période de sevrage thérapeutique de 2 mois (p 1,000 pour tous). La comparaison appariée de l’AIPE, de l’IELT, de l’IQR et de la SQOL masculine chez les patients du groupe (B) au départ et après le traitement par la duloxétine a montré une amélioration statistiquement significative chez tous les patients traités (*p* < 0,001 pour tous).

**Conclusions:**

La duloxétine est un médicament efficace pour traiter des patients atteints d’éjaculation précoce de longue durée, et ceux atteints d’éjaculation précoce acquise. D’autres études de plus grande envergure sont nécessaires pour comparer la duloxétine aux différentes modalités thérapeutiques connues de l’éjaculation précoce, afin de déterminer son efficacité et sa supériorité.

**Mots-clés:**

Ejaculation précoce de longue Durée; Ejaculation précoce acquise; Duloxétine; Qualité de Vie sexuelle masculine.

## Introduction

Premature ejaculation (PE) is considered to be the commonest male sexual disorder, affecting 20% to 66% of sexually active men [1–4]. Several definitions had described PE in the last few decades [1]. The international society for sexual medicine (ISSM) in 2014 had unanimously agreed that the items necessary to define PE are time from penetration to ejaculation, inability to delay ejaculation and negative personal consequences from PE [5]. The ISSM committee also agreed that ≤ 1 min IELT cutoff point should be considered for lifelong premature ejaculation (LPE), while reduced IELT to 3 min or less after a period of normal experience should be considered for acquired (secondary) premature ejaculation (APE) [5]. PE is mainly classified as lifelong (primary) (LPE) or acquired (secondary) (APE) [6]. Such classification was originally suggested by Waldinger & Schweitzer (2006) who proposed a new evidence based definition for PE through utilizing DSM-V and ICD 11 [7]. The exact aetiology of PE is unknown, with little data to support suggested biological and psychological hypotheses, including affective, cognitive, inter-personal or developmental psychological factors, penile hypersensitivity, prostatic congestion, chronic prostatitis and 5 hydroxytryptamine (5-HT) and dopamine receptor dysfunction [8]. However, Waldinger et al (1998) confirmed a genetic component for LPE [9]. Hormones might also play a role in the atieopathogensis of PE [6]. Additionally, some urological conditions might contribute to PE including prostatitis that causes APE [10, 11]. Interestingly, varicocele had been postulated to cause PE [12]. Duloxetine hydrochloride is an antidepressant included in the pharmacological class of serotonin (5-HT)-norepinephrine (NE) reuptake inhibitors (SNRIs) [13].

Pharmacokinetic and pharmacodynamic data of duloxetine had been reported for several studies, whose evidence suggested that duloxetine was generally well-tolerated [14]. However, duloxetine increases both levels of serotonin and norepinephrine which are directly correlated with adverse events including tachycardia and hypertension [15]. Hitherto, duloxetine is used as an off label therapy for PE. However, few studies showed that daily dose of duloxetine was an effective treatment option with very few adverse events in PE patients [16, 17]. Besides, a very recent study in China had demonstrated that dapoxetine was associated with a very high rate of discontinuation as results of over expectation of efficacy and high cost [18]. Henceforth, we aimed in the current study to assert the efficacy and safety profile of daily intake of 30 mg duloxetine in treating LPE patients and APE patients.

### Patients and methods

**Fig. 1:**
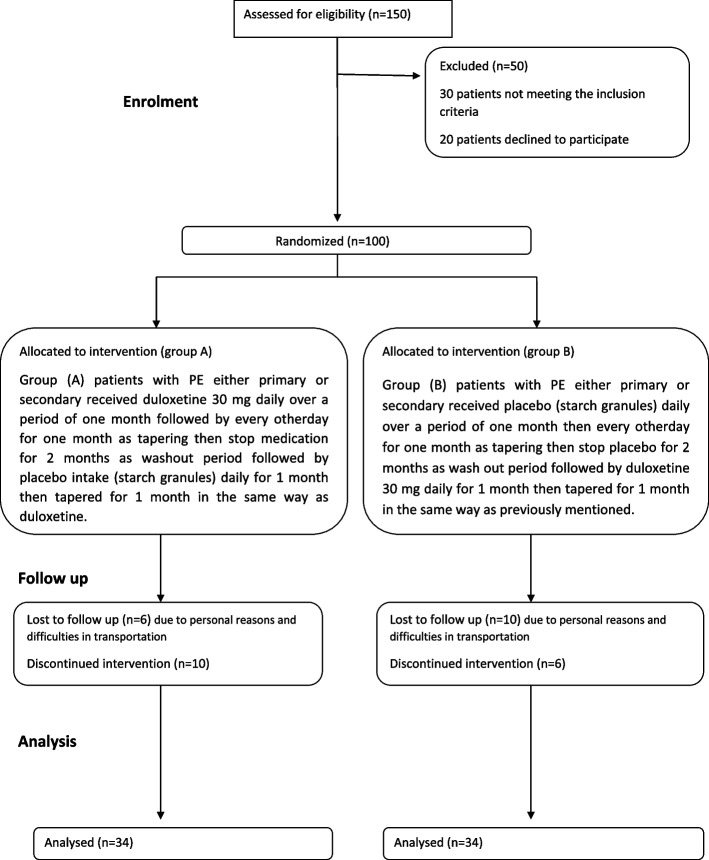
Shows a CONSORT flow chart diagram that represents the methodology of the study

The current randomized clinical trial assessed (150) patients for eligibility criteria with 50 patients excluded and 32 dropped out patients during the follow up period (Fig. 1). Finally, 68 patients having LPE and APE continued the study. The study was conducted in andrology outpatient clinic at Kasr Al-Ainy hospital from May 2022 to December 2022. The institutional review board had approved the work (MS-48–2022) that conforms to Helsiniki declaration (2013) [19]. All participants signed an informed consent prior to enrollment and were randomized by numbering method. The current study complied with the guidelines for reporting randomized parallel clinical trials (Fig. 1) [20].

#### Inclusion criteria of the patients

Male patients aged 25 to 45 years old, suffered from PE (primary or secondary) with stable and committed relationship having IELT < 3 min or less were included.

#### Exclusion criteria of the patients

Patients with erectile dysfunction (ED), reduced sexual desire or inhibited male orgasm or active genitourinary tract infection (confirmed by two glasses of urine and expressed prostatic secretions) or known mental disorders (depression, anxiety, schizophrenia) or uncontrolled physical illnesses (hepatic, renal, cardiac, neurological, endocrinal) were excluded from the study. Finally, any alcohol or drug abuser or patient with known hypersensitivity to duloxetine was excluded from the study.

Personal and past histories were taken from the participants. General and local examinations were done for all participants. Then patients were divided into two groups. Group (A) patients with LPE and APE received duloxetine 30 mg daily over a period of one month followed by every other day for another month as tapering then stop medication for 2 months as washout period followed by placebo intake (starch granules) daily for 1 month then tapered for another month in the same way as duloxetine. Duloxetine was prescribed after breakfast for all cases. However, cases who suffered from dizziness were advised to intake it after dinner. Group (B) patients with LPE and APE received placebo (starch granules) daily over a period of one month then every other day for another month as tapering then stop placebo for 2 months as wash out period followed by duloxetine 30 mg daily for 1 month then tapered for another month in the same way as previously mentioned.

Also, the validated Arabic version of the International index of erectile function (IIEF-5) was used initially to exclude ED [21]. Females were provided with a stopwatch that they received from andrology department and instructions on how to record the IELT. IELT that occurred before penile penetration into vagina was recorded as 0 min. The couples were required to experience a minimum of four coital attempts, with intervals of 3–7 days and to calculate a geometric mean IELT. They were instructed that if coitus took place more than once in a single session, only the first one would be recorded [22]. It was calculated using the following formula: IIELT = stopwatch IELT divided by half the male’s expected IELT + half the female’s expected IELT [22]. Also, the Arabic index of premature ejaculation (AIPE) was used initially to diagnose PE [23]. Furthermore, it was repeated after duloxetine and placebo to assess any change in the ejaculatory function [23]. Subjects were asked to rate their sexual desire, sexual potency, IELT time, their ability to delay IELT, sexual satisfaction to his partner [24]. Further, the level of stress or anxiety associated with sexual intercourse was also evaluated [24]. Additionally, the sexual quality of life men’s version (SQoL-M) was utilized in the current study initially and after duloxetine and placebo intake. The questionnaire has very good internal consistency as well as good test–retest reliability and convergent validity [25].

#### Statistical analysis

Statistical analysis was conducted using SPSS 22^nd^ edition (IBM Corp. Released 2013. IBM SPSS Statistics for Windows, Version 22.0. Armonk, NY: IBM Corp.), quantitative variables were presented in mean, SD for parametric data, and median, range for non-parametric data after normality testing using Shapiro–Wilk test.

Comparison of quantitative variables between groups was conducted using student T test for age, and Mann Whitney U test all scores assessed in the current study, paired comparison of assessed scores was conducted using Wilcoxon sign rank test. Categorical variables were presented in frequency and percentages and compared using Chi square test. Any *p* value < 0.05 was considered significant.

#### Sample size determination

Clinical sample size calculator was used; with 0.05 alpha error and power of the study 0.80 with 1:1 enrollment ratio to calculate minimal sample size needed to detect effect of duloxetine (30 mg) in treating PE. The total sample size calculated was 62 PE patients (31 in each arm of the study). A random sample of PE patients with inclusion and exclusion criteria would be assigned to the study. Each individual would be randomized and allocated to either intervention arm: duloxetine 30 mg treatment daily in the first month and followed by 30 mg every other day in the next month then washout for 2 months and then cross over to placebo or control arm: placebo for 2 months then washout 2 months and cross over to duloxetine 30 mg. Thus, a concealed random allocation method was adopted till reaching the required total sample size.

## Results

The mean age of patients was 38.4 ± 6.1 years. APE accounted for 91.2%. Sociodemographic characteristics are shown in Table 1. Comparison after 2 months from randomization, treatment group showed statistically significant median AIPE score 26 versus 19.5, longer duration of median IELT 180 s versus 60 s and higher median male SQOL score 45 vs 21 (*p* value < 0.001 for all) (Table 2).

**Table 1:** Shows sociodemographic characteristics of participants

Participants (*N* = 68)	Median
IIEF-5^a^	24
baseline	
AIPE^b^	19
IELT (Sec)^c^	60
Male SQOL^d^	20
AIPE after duloxetine^b^	26
IELT after duloxetine (Sec)^c^	180
Male SQOL after duloxetine^d^	43
AIPE after placebo^b^	19
IELT after placebo (Sec)^c^	60
Male SQOL after placebo^d^	21

^a^*IIEF-5*  the international index of erectile function

^b^*AIPE*  the Arabic index of premature ejaculation

^c^*IELT*  Intravaginal ejaculatory latency time

^d^Male SQOL (sexual quality of life questionnaire- male version) before and after treatment in both the placebo and the treatment groups. Chi2 test was used in this table

**Table 2:** Compares between baseline characteristics and effect of treatment after 2 months among participants

	Group						*P* value
	A			B			
	Median	Minimum	Maximum	Median	Minimum	Maximum	
IIEF-5^a^	24	22	33	24	22	25	0.621^e^
AIPE baseline^b^	19	9	20	19.5	8	30	0.41^e^
IELT baseline (Sec)^c^	60	30	120	60	30	120	0.577^e^
Male SQOL baseline^d^	20	15	30	21	16	33	0.294^e^
AIPE after treatment^b^	26	9	34	19.5	8	30	< 0.001^e^
IELT after treatment (Sec)^c^	180	30	240	60	30	120	< 0.001^e^
Male SQOL after treatment^d^	45	18	55	21	16	33	< 0.001^e^

^a^*IIEF-5* the international index of erectile function

^b^*AIPE* the Arabic index of premature ejaculation

^c^*IELT* Intravaginal ejaculatory latency time

^d^Male SQOL (sexual quality of life questionnaire- male version) before and after treatment in both placebo and treatment groups

^e^*P* value was calculated using Mann Whitney U test

Comparison between study groups in terms of baseline AIPE, baseline IELT, baseline male SQOL showed no statistically significant difference between study groups (Table 2). However, group (A) patients revealed a statistically significant male SQOL compared to group (B) patients (*p* value = 0.029) (Table 3). Paired comparison of AIPE, IELT (Sec), IQR and male SQOL in group (A) patients at baseline and after duloxetine intake showed statistically significant improvement among treated patients (*p* values < 0.001 all) (Table 4). Paired comparison of AIPE, IELT (Sec), IQR and male SQOL in group (A) patients at baseline and after placebo intake showed no significant improvement of male SQOL (Table 4). Furthermore, AIPE and IELT returned to baseline scores after discontinuation of duloxetine (*p* values 0.729; 0.892, respectively) (Table 4). Paired comparison of AIPE, IELT (Sec), IQR and male SQOL in group (B) patients at baseline and after placebo intake showed almost same scores of patients in group (A) who received placebo for 2 months after a 2 month washout period (*p* values 1.000 for all) (Table 5). Paired comparison of AIPE, IELT (Sec), IQR and male SQOL in group (B) patients at baseline and after duloxetine intake showed statistically significant improvement among all treated patients (*p* values < 0.001 all) (Table 5). It is worth mentioning that none of our patients stopped the drug due to side effects as we used only 30 mg. However, it should be noted that mild dizziness forced some patients to intake the drug after dinner instead of breakfast.

**Table 3:** Compares between study groups after cross over

	Group						*P* value
	A			B			
	Median	Minimum	Maximum	Median	Minimum	Maximum	
AIPE Baseline^a^	19	9	30	19.5	8	30	0.41^d^
IELT baseline (Sec)^b^	60	30	120	60	30	120	0.577^d^
Male SQOL baseline^c^	20	15	30	21	16	33	0.294^d^
AIPE after duloxetine^a^	26	9	34	26	14	34	0.626^d^
IELT after duloxetine (Sec)^b^	180	30	240	240	120	240	0.283^d^
Male SQOL after duloxetine^c^	45	18	55	40	30	55	0.029^d^
AIPE after placebo^a^	19	9	30	19.5	8	30	0.995^d^
IELT after placebo (Sec)^b^	60	30	160	60	30	120	0.292^d^
Male SQOL after placebo^c^	21	17	30	21	16	30	0.617^d^

^a^*AIPE* The Arabic index of premature ejaculation

^b^*IELT* Intravaginal ejaculatory latency time

^c^*Male SQOL* (sexual quality of life questionnaire- male version) after cross over

^d^*P* value was calculated using Mann Whitney U test

**Table 4:** Shows a paired comparison of baseline scores and post duloxetine and post placebo in group A

Group A	Baseline		After duloxetine		*p*-value	Baseline		After placebo		*p*-value
	Median	IQR	Median	IQR		Median	IQR	Median	IQR	
AIPE ^a^	19	9–30	26	9–34	< 0.001	19	15–22	19	17–24	0.729^d^
IELT (Sec)^b^	60	30–120	180	30–240	< 0.001	60	30–60	60	30–60	0.892^d^
Male SQOL^c^	20	15–30	45	18–55	< 0.001	20	18–21	21	20–22	0.865^d^

^a^*AIPE* the Arabic index of premature ejaculation

^b^*IELT* Intravaginal ejaculatory latency time

^c^*Male SQOL* (sexual quality of life questionnaire- male version) before and after treatment group A

^d^*P* value was calculated using Wilcoxon sign rank test

**Table 5:** Shows a paired comparison of baseline scores and post duloxetine and post placebo in group B

Group B	Baseline		After placebo		*p*-value	Baseline		After duloxetine		*p*-value
	Median	IQR	Median	IQR		Median	IQR	Median	IQR	
AIPE^a^	19.5	8–30	19.5	8–30	1.000	19.5	8–30	26	14–34	< 0.001^d^
IELT(Sec)^b^	60	30–120	60	30–120	1.000	60	30–120	240	120–240	< 0.001^d^
Male SQOL^c^	20	16–34	21	16–33	1.000	21	16–33	40	30–55	< 0.001^d^

^a^*AIPE* the Arabic index of premature ejaculation

^b^*IELT* Intravaginal ejaculatory latency time

^c^Male SQOL (sexual quality of life questionnaire- male version) before and after treatment group B

^d^*P* value was calculated using Wilcoxon sign rank test

## Discussion

A total of 68 patients continued the study where the majority of them did not improve on dapoxetine prior to joining the current study. Before inclusion, all patients were asked to measure their IELT for a month using a stop-watch. The median IELT of the patients in our study was 60 s.

The median AIPE in the current study was 19 that considered as moderate severity. Moreover, the median male SQOL was 20 indicating a low quality of life. The low baseline data showed significant improvement after daily intake of 30 mg duloxetine over one month then followed by every other day tapering for another month in the two groups. Notably, 32 patients from the two groups were lost to follow up. Consistently, when the two groups compared together after 2 months of duloxetine intake, they revealed statistically significant AIPE, longer duration of IELT and higher male SQOL score. Dizziness was the main side effect that reported by all patients treated with duloxetine in the current study. In light of the current knowledge, few studies assessed the role of duloxetine in PE. Athanasios et al. (2007) demonstrated that duloxetine was more competent than placebo in treating PE when administered on chronic basis as well as a major improvement in psychological parameters determined by clinical global impression-improvement Scale (CGI-I) [16]. In the same line, Ozcan et al. (2015) had shown that duloxetine was safe and effective for treating for PE [17]. PE was associated with increased anxiety and depression, frustration, anger and loss of sexual confidence [26]. Consistently, a recent Egyptian study by Hanafy et al. (2019) revealed a significant affection of the QoL in PE patients [27]. Henceforth, it is important to evaluate the sexual quality of life in PE patients as in the current study, the male SQOL significantly improved after duloxetine intake for 2 months in the two groups. Interestingly, group (A) patients who started duloxetine first showed significant improvement in their sexual quality of life compared to group (B) patients who started placebo first. Thus, patients who started duloxetine first felt such improvement compared to group (B) who continued to suffer for 4 months before receiving duloxetine. In the same context, Athanasios et al. (2007) and Ozcan et al. (2015) revealed similar findings [16, 17].

In contrast, Lui et al. (2020) conducted a systemic meta-analysis that showed that drug combination of selective serotonin reuptake inhibitor plus phosphodiestrase type 5 inhibitor was the most effective PE therapy on chronic basis [28]. However, the favourable effects of duloxetine on PE demonstrated in the current study can be explained by several facts. Firstly, in vitro experimental studies had shown that duloxetine preferentially inhibits 5-HT reuptake more than NE reuptake [29, 30]. Furthermore, chronic duloxetine administration has a long-term modulatory effect on 5-HT and NE pathways without an effect on basal 5-HT, NE, or dopamine levels in the cerebral cortex, a moderate effect on 5-HT and NE release in the hippocampus and a substantial desensitization of terminal α2-heteroreceptors but not 5-HT1B receptors [31]. Thus, it can be suggested that chronic duloxetine administration results in adaptive changes of autoreceptor functions equivalent to chronic administration of selective serotonin reuptake inhibitors and noradrenaline reuptake inhibitors [32]. However, such adaptive changes fail to reset 5-HT and NE levels, respectively [32].

### Limits of the study

Admittedly, small sample size and inability to follow up the cases for longer duration can be seen as the major limitation of the study. Also, the utility of AIPE for diagnosing cases of PE can be added as another limitation as it is of little clinical use. Besides, sexual quality of life in all participants was evaluated by a questionnaire that was not validated in Arabic. Moreover, inclusion of LPE and APE in the same group can be considered as another limitation of the study. Furthermore, all cases of APE suffered from IELT < 2 min and not < 3 min can be added for the limitations of the study. Finally, there was a significant loss of follow up that was attributed mainly to difficult transportation as most patients were coming from far areas.

Thus, it could be repharsed that an intuition-to-treat analysis was performed and maybe the lost patients were the ones that were unhappy with treatment.

## Conclusion

A daily intake of duloxetine 30 mg had an effective role in decreasing PE severity and improving sexual quality of life in LPE and APE patients. Larger studies are needed to assert it’s superiority compared to other known therapeutic modalities for PE.

## Data Availability

The data that support the findings of this study are available from the corresponding author upon reasonable request.

## Abbreviations

IELT: Intra-vaginal ejaculation latency time
SNRIs: Serotonin (5-HT)-norepinephrine (NE) reuptake inhibitors
IIEF-5: The international index of erectile function
AIPE: The Arabic index of premature ejaculation
ED: Erectile dysfunction
Male SQOL: Sexual quality of life questionnaire- male version
APE: Acquired premature ejaculation
LPE: Lifelong premature ejaculation
5-HT: 5 Hydroxytryptamine
